# Quantitative Proteomic Analysis of Outer Membrane Vesicles from Fusobacterium nucleatum Cultivated in the Mimic Cancer Environment

**DOI:** 10.1128/spectrum.00394-23

**Published:** 2023-06-21

**Authors:** Xuqiang Zhang, Yuxin Wang, Ruochen Fan, Liying Zhang, Zhuting Li, Yanmei Zhang, Wei Zheng, Lulu Wang, Baoquan Liu, Chunshan Quan

**Affiliations:** a Key Laboratory of Biotechnology and Bioresources Utilization of the Ministry of Education, College of Life Science, Dalian Minzu University, Dalian, Liaoning, China; b Department of Bioengineering, College of Life Science, Dalian Minzu University, Dalian, Liaoning, China; c School of Life Science and Biotechnology, Dalian University of Technology, Dalian, Liaoning, China; Institut National de Santé Publique du Québec

**Keywords:** outer membrane vesicles, *Fusobacterium nucleatum*, LC-MS/MS, tandem mass tag, virulence proteins

## Abstract

Fusobacterium nucleatum is a Gram-negative bacterium that has been identified as an important pathogenic gut bacterium associated with colorectal cancer. Compared with the normal intestine, the pH value of the tumor microenvironment is weakly acidic. The metabolic changes of F. nucleatum in the tumor microenvironment, especially the protein composition of its outer membrane vesicles, remain unclear. Here, we systematically analyzed the effect of environmental pH on the proteome of outer membrane vesicles (OMVs) from F. nucleatum by tandem mass tag (TMT) labeling–high-resolution liquid chromatography-tandem mass spectrometry (LC-MS/MS) analysis. A total of 991 proteins were identified in acidic OMVs (aOMVs) and neutral OMVs (nOMVs), including known virulence proteins and putative virulence proteins. Finally, 306 upregulated proteins and 360 downregulated proteins were detected in aOMVs, and approximately 70% of the expression of OMV proteins was altered under acidic conditions. A total of 29 autotransporters were identified in F. nucleatum OMVs, and 13 autotransporters were upregulated in aOMVs. Interestingly, three upregulated autotransporters (D5REI9, D5RD69, and D5RBW2) show homology to the known virulence factor Fap2, suggesting that they may be involved in various pathogenic pathways such as the pathway for binding with colorectal cancer cells. Moreover, we found that more than 70% of MORN2 domain-containing proteins may have toxic effects on host cells. Gene Ontology (GO) and Kyoto Encyclopedia of Genes and Genomes (KEGG) enrichment analyses demonstrated that a number of proteins were significantly enriched in multiple pathways involving fatty acid synthesis and butyrate synthesis. Seven metabolic enzymes involved in fatty acid metabolism pathways were identified in the proteomic data, of which 5 were upregulated and 2 were downregulated in aOMVs, while 14 metabolic enzymes involved in the butyric acid metabolic pathway were downregulated in aOMVs. In conclusion, we found a key difference in virulence proteins and pathways in the outer membrane vesicles of F. nucleatum between the tumor microenvironment pH and normal intestinal pH, which provides new clues for the prevention and treatment of colorectal cancer.

**IMPORTANCE** F. nucleatum is an opportunistic pathogenic bacterium that can be enriched in colorectal cancer tissues, affecting multiple stages of colorectal cancer development. OMVs have been demonstrated to play key roles in pathogenesis by delivering toxins and other virulence factors to host cells. By employing quantitative proteomic analysis, we found that the pH conditions could affect the protein expression of the outer membrane vesicles of F. nucleatum. Under acidic conditions, approximately 70% of the expression of proteins in OMVs was altered. Several virulence factors, such as type 5a secreted autotransporter (T5aSSs) and membrane occupation and recognition nexus (MORN) domain-containing proteins, were upregulated under acidic conditions. A large number of proteins showed significant enrichments in multiple pathways involving fatty acid synthesis and butyrate synthesis. Proteomics analysis of the outer membrane vesicles secreted by pathogenic bacteria in the acidic tumor microenvironment is of great significance for elucidating the pathogenicity mechanism and its application in vaccine and drug delivery vehicles.

## INTRODUCTION

The Gram-negative anaerobic bacterium Fusobacterium nucleatum has attracted considerable attention recently. It is isolated most frequently from both healthy and diseased sites in the oral cavity and is commonly known as a key pathogen in gingivitis and periodontitis (1–3). It has also been isolated in several inflammatory processes at distinct body sites, such as endocarditis (4, 5), septic arthritis (6, 7), liver (8, 9) and brain (10, 11) abscesses and has been implicated in adverse pregnancy outcomes (12, 13). F. nucleatum was first reported in colorectal cancer (CRC) tissue in 2011, suggesting that a higher abundance of F. nucleatum was significantly associated with CRC (14, 15). In recent years, a number of articles and data have also shown that the abundance of F. nucleatum in tumor tissues and feces of patients with colorectal cancer is significantly higher than that in the normal population, which can promote the occurrence and development of colorectal cancer and is closely related to its recurrence and poor prognosis (16–18). There is evidence that F. nucleatum participates in mechanisms of inflammation (19–21), immune regulation (22), genotoxin production (2, 23, 24), and the production of metabolites that are harmful to the intestinal epithelium (25).

Outer membrane vesicles (OMVs) are spherical double-layered nanoparticles ranging from 50 to 250 nm in diameter (26) that are considered ubiquitous across all areas of life and include all internal bacterial cell components (virulence factors, enzymes, DNA, lipids, sugars, and communication signals) (27–35). Several bacterium-derived OMVs, even in the absence of any living bacteria, have demonstrated cytotoxicity due to their virulence factors such as leukotoxin, adenylate cyclase toxin, and hemolysin during bacterial infections. Additionally, OMVs play essential roles in drug resistance and intercellular communication. Zhang et al. demonstrated previously that the outer membrane vesicles derived from hypervirulent Klebsiella pneumoniae (hvKP) contain a variety of proteins and exhibit different cytotoxic effects on different cell types *in vitro* and that the outer membrane vesicles induce the expression of proinflammatory cytokines in host cells, including interleukin-6 (IL-6) and IL-8 (36).

Previous studies have shown that F. nucleatum subsp. *nucleatum*, F. nucleatum subsp. *polymorphum*, and F. nucleatum subsp. *animalis* can secrete OMVs. Liu et al. previously identified 98 proteins from FnOMVs (OMVs from F. nucleatum) by proteomic techniques, among which 6 autotransporters were associated with the V-type secretion system and virulence factor proteins with functional domains, including FadA, MORN2, and YadA-like domains (37). FnOMVs trigger the innate immunity of human intestinal epithelial cells by promoting NF-κB activation via dynamin-mediated endocytosis, and FomA is involved in the NF-κB response (38). In macrophage/Caco-2 cocultures, FnOMVs significantly promoted epithelial barrier loss and oxidative stress damage by activating the RIPK1-mediated epithelial cell death pathway (39).

Intestinal pH is an important physiological condition for maintaining the growth of intestinal microorganisms and the digestion and absorption of nutrients (40). Under normal physiological conditions, the extracellular pH is usually maintained in the range of pH ~6.9 to 7.5. The acidic microenvironment caused by massive anaerobic glycolysis is an important characteristic of malignant tumors and is an important factor in inducing tumorigenesis, metastasis, and drug resistance. The external pH of cancer tissue decreases due to changes in the microenvironment caused by cancer and is usually between 6.0 and 6.8 (41, 42). Under different pH conditions, the metabolic activities of bacterial cells are affected, resulting in differences in protein expression and the protein composition of the outer membrane vesicles. At present, the complete composition and biological function of outer membrane vesicles from F. nucleatum remain unclear, although partial proteomic and functional analyses of the outer membrane vesicles of F. nucleatum were performed by Liu et al. (37), and the composition and function of outer membrane vesicles secreted by bacteria in the tumor environment have not been reported.

In the present study, we performed a comprehensive proteomic analysis of F. nucleatum outer membrane vesicles at the tumor microenvironment pH and F. nucleatum outer membrane vesicles at normal intestinal pH using tandem mass tag (TMT) quantitative proteomics techniques. The levels of 666 proteins (306 up- and 360 downregulated proteins) in the outer membrane vesicles were significantly different between the two pH conditions, and approximately 70% of the expression of OMV proteins was altered under acidic conditions. GO and KEGG enrichment analyses demonstrated that a number of proteins showed significant enrichments in multiple pathways involving fatty acid synthesis and butyrate synthesis. Additionally, changes in the pH affected the expression of virulence factors. Seven type 5a secreted autotransporter (T5aSSs) and 16 membrane occupation and recognition nexus (MORN) domain-containing proteins were upregulated in acidic OMVs (aOMVs), suggesting that these functional proteins and related pathways may play important physiological and biochemical roles in the tumor microenvironment.

## RESULTS

### Characteristics of F. nucleatum OMVs under two different pH conditions.

Two typical ecological niches in which F. nucleatum colonizes the colorectum are the normal intestinal environment (pH 7.0) and the tumor environment (pH 6.0). In the gut, F. nucleatum produces, secretes, and utilizes OMVs as virulence factors that cause intestinal inflammation and promote the formation of a tumor microenvironment suitable for survival. The formation of the tumor environment is affected by pathogenic bacteria such as F. nucleatum, and it also affects the physiological activity and virulence of F. nucleatum. To investigate the effects of different ecological niches on the production and toxicity of OMVs, F. nucleatum was cultivated in medium at pH 7.0 and pH 6.0 to mimic the normal intestinal pH environment and the tumor pH environment, respectively. For convenience, OMVs secreted under pH 7.0 conditions are referred to as neutral outer membrane vesicles (nOMVs), and those secreted under pH 6.0 conditions are referred to as acidic outer membrane vesicles (aOMVs).

**FIG 1:**
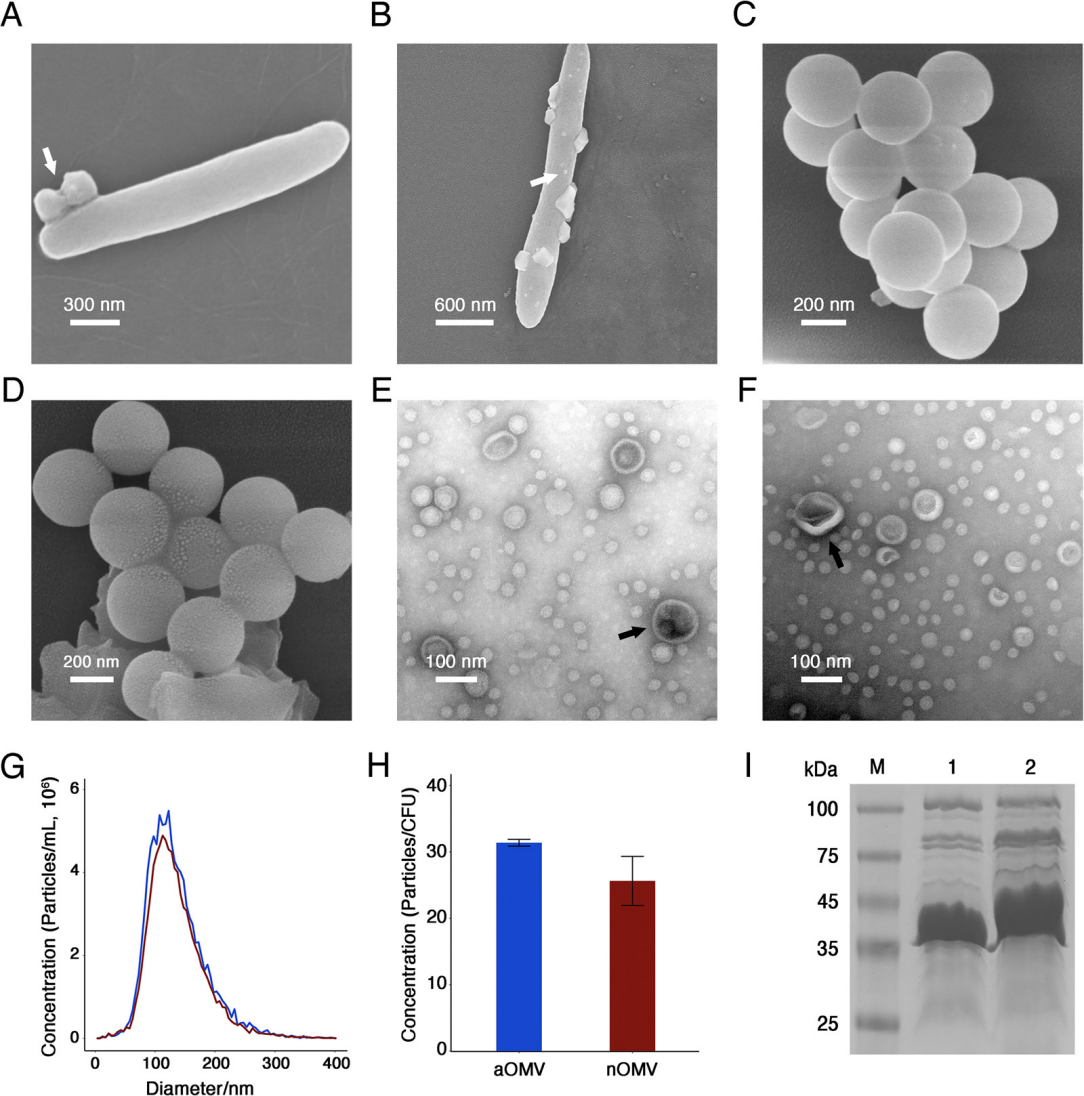
Characteristics of OMVs produced by F. nucleatum ATCC 23726 under different pH conditions. (A) SEM image of F. nucleatum and OMVs (white arrow) under neutral conditions. Magnification, ×500,000. (B) SEM image of F. nucleatum and OMVs (white arrow) under acidic conditions. Magnification, ×300,000. (C) SEM image of purified nOMVs. Magnification, ×600,000. (D) SEM image of purified aOMVs. Magnification, ×600,000. (E) TEM image of purified nOMVs. Magnification, ×600,000. (F) TEM image of purified aOMVs. Magnification, ×600,000. (G) Size distribution of F. nucleatum OMVs. Red line, nOMVs; blue line, aOMVs. (H), Concentration analysis of OMVs. Error bars represent standard deviations. (I) SDS-PAGE analysis of OMVs. Lane M, marker; lane 1, aOMVs; lane 2, nOMVs.

The production of nOMVs and aOMVs was detected by scanning electron microscopy (SEM). Under both conditions, prominent spherical particles appeared on the surface of F. nucleatum cells (Fig. 1A and B), indicating that the strain could release OMVs at both pH values. To study the effects of pH on F. nucleatum OMVs, OMVs were isolated and purified from the culture supernatant of F. nucleatum by ultracentrifugation and density gradient centrifugation. SEM and transmission electron microscopy (TEM) results showed that both nOMVs (Fig. 1C to E) and aOMVs (Fig. 1D to F) were spherical particles of different sizes, some particles in the field of view showed a double-layer film, and the structure of aOMVs also showed a cup-like structure. Overall, the pH had no significant effect on the morphology of the OMVs. Nanoparticle tracking analysis (NTA) revealed that the particle size of aOMVs was 136.2 ± 50.3 nm, with an average value of 135.5 ± 48.5 nm, and the aOMVs were concentrated mainly in the particle size range of 83.0 to 194.5 nm (Fig. 1G). There was no significant difference between the particle sizes of nOMVs and aOMVs, and the pH had little effect on the particle size of OMVs. As shown in Fig. 1H, the yield of nOMVs was 26 ± 3 particles/CFU, and the yield of aOMVs was 32 ± 1 particles/CFU. The level of OMV production at pH 6.0 was 1.23 times higher than that at pH 7.0. The effects of pH on the protein composition of the F. nucleatum OMVs were preliminarily analyzed by SDS-PAGE. As shown in Fig. 1I, aOMVs (lane 1) have a broad band between 35 kDa and 45 kDa and a few specific bands near 50 kDa and 75 kDa. However, nOMVs (Fig. 1I, lane 2) have a broad band between 35 kDa and 50 kDa and specific bands near 65 kDa, 75 kDa, and 80 kDa. This indicates that pH may affect the protein composition of F. nucleatum OMVs.

### Identification of proteins in F. nucleatum OMVs.

Intestinal pH is an important physiological condition for maintaining the growth of intestinal microorganisms and the digestion and absorption of nutrients by the human body. Compared with the normal intestine, the pH value of the environment surrounding a tumor is significantly lower. To explore the changes in the protein composition and function of F. nucleatum OMVs caused by the pH reduction in the intestinal tumor environment, liquid chromatography-tandem mass spectrometry (LC-MS/MS) proteomics with tandem mass tags (TMTs) was used to quantify the protein levels in aOMVs and nOMVs. Experimental repeatability was assessed using two statistical methods: the distributions for the normalized scaled intensity of proteins and Pearson’s correlation coefficient. A violin plot (see Fig. S1A in the supplemental material) shows that the medians of the samples in the same group are close to the same horizontal line, and the Pearson correlation coefficient (Fig. S1B) between aOMVs and nOMVs is approximately 1, indicating that the samples within the group are well reproducible.

**FIG 2:**
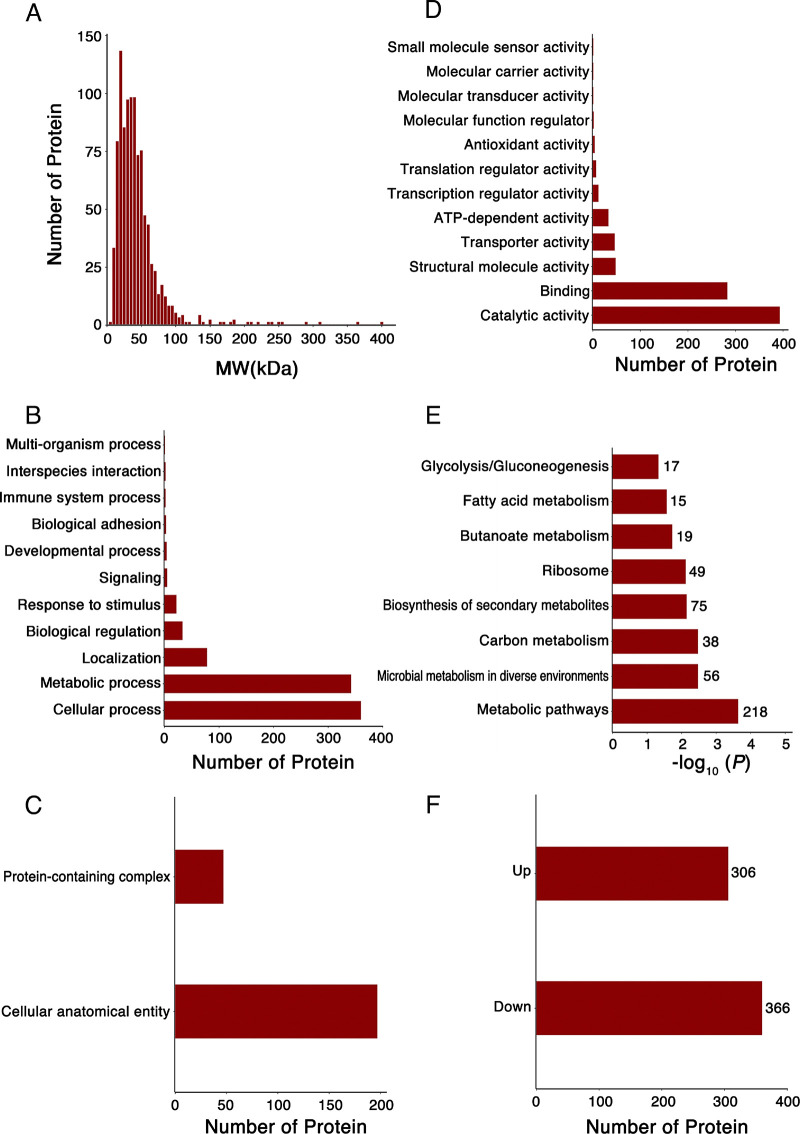
Quantitative analysis of the 991 proteins common to both aOMVs and nOMVs. (A) Protein molecular weight (MW) distribution. (B to D) GO enrichment analysis of the 991 proteins in terms of biological processes (B), cellular components (C), and molecular functions (D). Bars represent the matched protein counts. (E) KEGG enrichment analysis of the 991 proteins. (F) Statistical distribution of DEPs. The numbers to the right of the bars indicate the numbers of proteins identified.

A total of 549,010 MS spectra were obtained by mass spectrometry from three parallel TMT experiments (Table S1), and a total of 78,986 mass spectra were matched with known mass spectra with an ~14.4% spectrum utilization rate. Totals of 14,039 unique peptides and 991 quantifiable proteins were identified (Table S2). The length distribution of the peptides indicates that most of them consist of 6 to 20 amino acids (Fig. S1C). As shown in Fig. 2A, proteins with relative molecular weights of 30 to 40 kDa were the most abundant, followed by proteins with relative molecular weights of 15 to 30 kDa and 40 to 55 kDa. Based on their localizations, the identified proteins were classified into three groups: the cell membrane region (99 proteins), the cytoplasm region (45 proteins), and ribosomes (47 proteins) (Fig. S1D). GO analysis grouped the results according to biological processes (BPs), cellular components (CCs), and molecular functions (MFs). In terms of biological processes, F. nucleatum OMV proteins were involved mainly in cellular processes, metabolic processes, and biological regulation (Fig. 2B). In terms of cellular components, F. nucleatum OMV proteins were enriched in cellular anatomical entities and protein-containing complexes (Fig. 2C). In terms of molecular functions, F. nucleatum OMV proteins were enriched in molecular binding and catalytic activities (Fig. 2D). The results of KEGG enrichment analysis showed that the identified proteins exhibited a broad functional distribution and were involved mainly in various metabolic pathways and biosynthetic pathways for secondary metabolites (Fig. 2E). To further analyze the effects of pH on the protein level and metabolic function of F. nucleatum OMVs, protein differential expression analysis was performed. In the statistical analysis of differentially enriched proteins, a *P* value of <0.05 and 1/1.2 < FC (fold change) <1.2 were used as the statistical standards for differentially expressed proteins (DEPs). Based on these two criteria, 666 DEPs were isolated (Table S3), of which 360 were significantly downregulated and 306 were significantly upregulated (Fig. 2F), indicating that pH affects the protein composition and metabolic function of F. nucleatum OMVs.

### Differential proteomics analysis.

**FIG 3:**
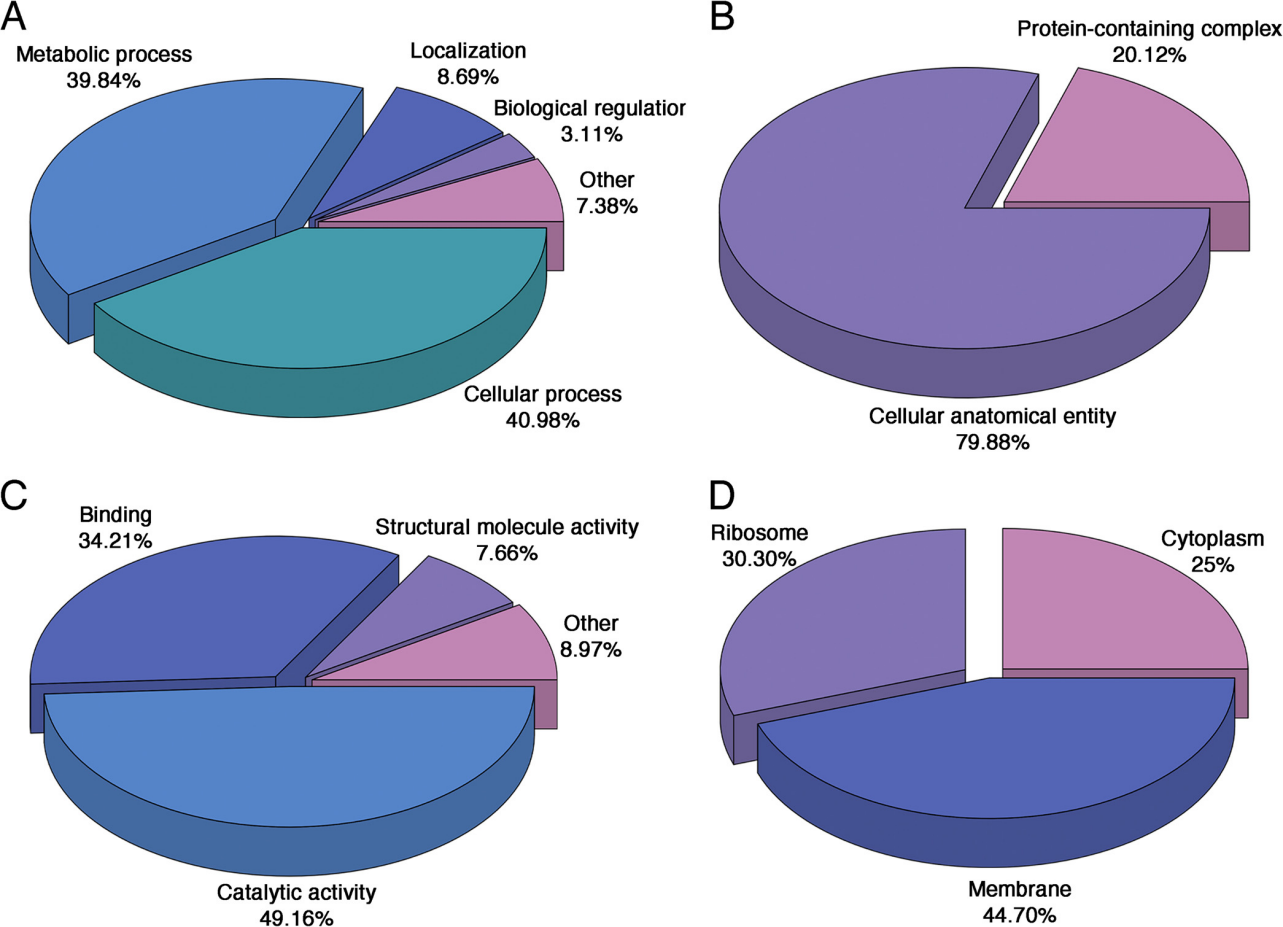
GO functional classification and subcellular structural localization classification of identified differentially enriched proteins based on biological processes (A), cellular components (B), molecular functions (C), and subcellular localization (D).

Differential proteomics analysis promotes the discovery of new drug targets and biomarkers, which can improve the accuracy of targeting and recognition, and is of great value for the prevention and treatment of clinical diseases. GO function analysis was performed on all differentially expressed proteins in the aOMV/nOMV comparison group. The differentially expressed proteins were involved mainly in three categories: biological processes, cellular components, and molecular functions. In the biological process category (Fig. 3A), most proteins were involved mainly in cellular processes (40.98%) and metabolic processes (39.84%). In the cellular component category (Fig. 3B), the differentially expressed proteins were distributed mainly in cellular anatomical entities (79.88%) and protein-containing complexes (20.12%). In the molecular function category (Fig. 3C), the differentially expressed proteins were related mainly to catalytic activity (49.16%) and binding processes (34.21%). The results of protein subcellular structure localization analysis (Fig. 3D) showed that most of the differentially expressed proteins were localized on the cell membrane (44.70%), followed by ribosomes (30.30%), and only 25% were localized in the cytoplasm.

**FIG 4:**
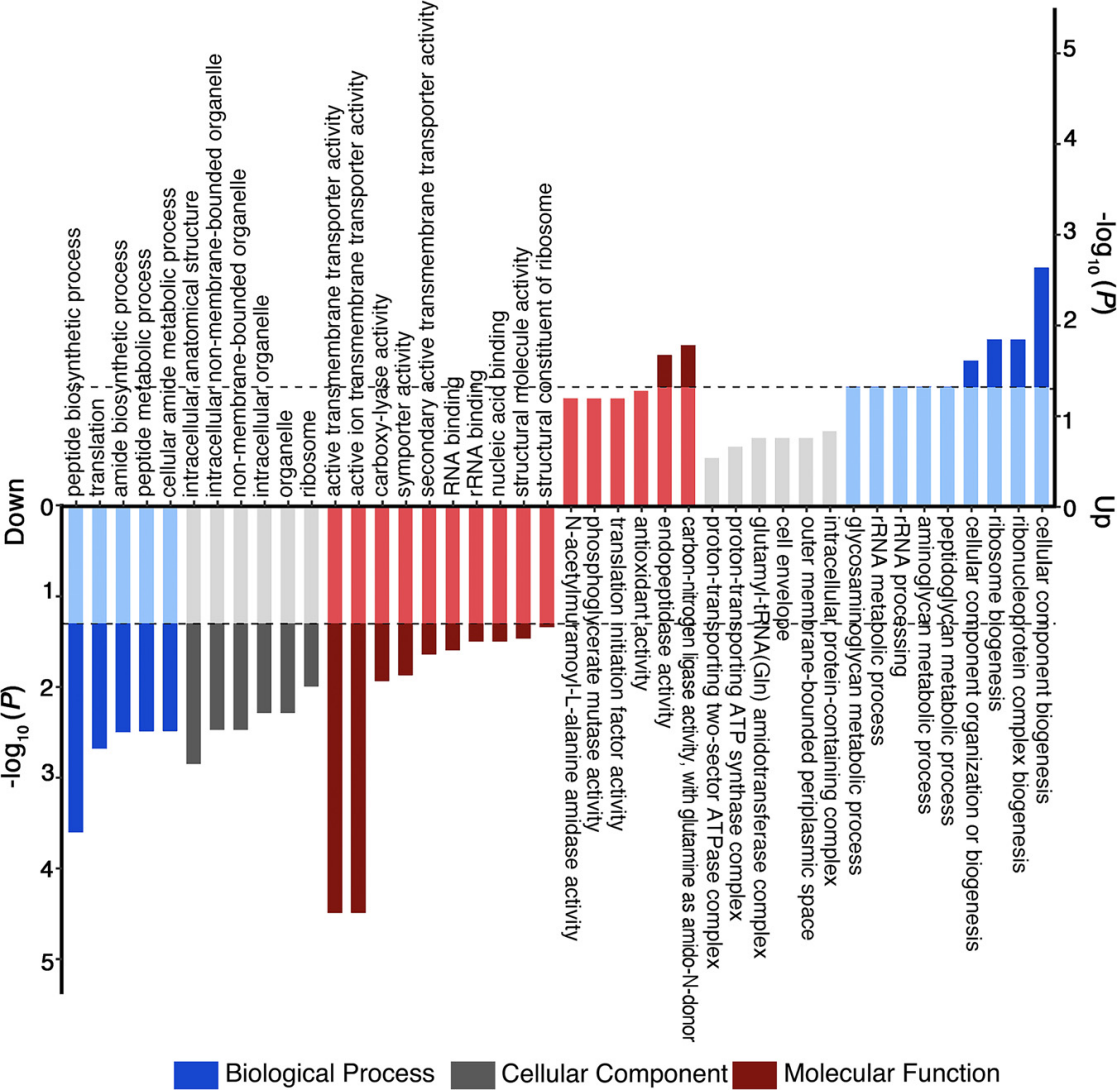
Analysis of molecular functions, cellular components, and biological processes of upregulated proteins and downregulated proteins based on GO enrichment. The dotted lines represent log_10_(*P* values) of 0.05. The vertical axis value is a negative logarithmic transformation of significant *P* values (*P* values of <0.05).

GO functional enrichment analysis of DEPs was also conducted based on the annotation data for aOMV/nOMV whole proteins (Fig. 4). The horizontal gray line in Fig. 4 represents the −log_10_ cutoff at a *P* value of 0.05. At the biological process level, the upregulated proteins were concentrated mainly in the biogenesis of cell components, ribonucleoprotein complex biogenesis, and ribosome biogenesis, while the processes of cellular amide metabolism and peptide metabolism showed a downward trend. From the perspective of cellular components, most of the differentially expressed proteins were downregulated, and the downregulated proteins belonged mainly to cytoplasmic ribosomal components and some organelle components. At the molecular function level, the upregulated proteins were enriched mainly in proteins with carbon-nitrogen ligase activity and endopeptidase activity and related functional proteins, while the downregulated proteins were concentrated mainly in ribosomal structural component-related proteins and structural molecular activity-related functional proteins.

In the aOMV/nOMV comparison group, the significantly upregulated differentially expressed proteins were located in several KEGG pathways. Analysis of the metabolic pathways of these differentially expressed proteins could help us better understand the effects of pH changes on the metabolism and pathogenicity of F. nucleatum in the tumor environment. KEGG pathway enrichment analysis revealed that the upregulated differentially expressed proteins were significantly enriched in fatty acid biosynthesis pathways. The fatty acid synthesis pathway is an important basal metabolic pathway of F. nucleatum and is closely related to phospholipid synthesis, cell membrane assembly, cell signal transduction, energy storage, and gene expression regulation. The fatty acid synthesis pathway (Fig. S2) is divided into two stages: synthesis initiation and carbon chain elongation. In the initial stage of fatty acid synthesis, malonyl-CoA is first synthesized from acetyl-CoA by acetyl-CoA carboxylase carboxyltransferase, and acyl carrier protein (ACP) is then transferred to malonyl-CoA by *S*-malonyltransferase to generate malonyl-ACP. Compared with nOMVs, both the α-subunit (AccA) and the β-subunit (AccD) of acetyl-CoA carboxylase carboxyltransferase were downregulated in aOMVs. It is suggested that the amount of malonyl-ACP may be reduced when entering the carbon chain elongation stage because the quantity and function of the key enzyme acetyl-CoA carboxylase carboxyltransferase are affected. Entering the carbon chain extension stage, four reactions are required to complete each round of carbon chain extension, namely, condensation, reduction, dehydration, and reduction. In each chain extension cycle, 2 carbons are added until long-chain fatty acids of 16 to 18 carbon atoms are formed. Among them, FabH and FabF participate in the condensation reaction, FabG participates in the reduction reaction of acetoacetyl-ACP, FabZ catalyzes the dehydration reaction, and FabI finally reduces *trans*-2,3-dehydroacyl-ACP to acyl-ACP and enters a new cycle reaction. All of the enzymes involved in the carbon chain extension stage were upregulated in aOMVs, suggesting that acidic pH conditions are favorable for the synthesis of long-chain fatty acids. The growth pH of F. nucleatum significantly affects fatty acid anabolism. To adapt to the pH changes in the growth environment, cells adjust the fatty acid composition to change the permeability of the phospholipid bilayer to minimize energy consumption and optimize growth.

**FIG 5:**
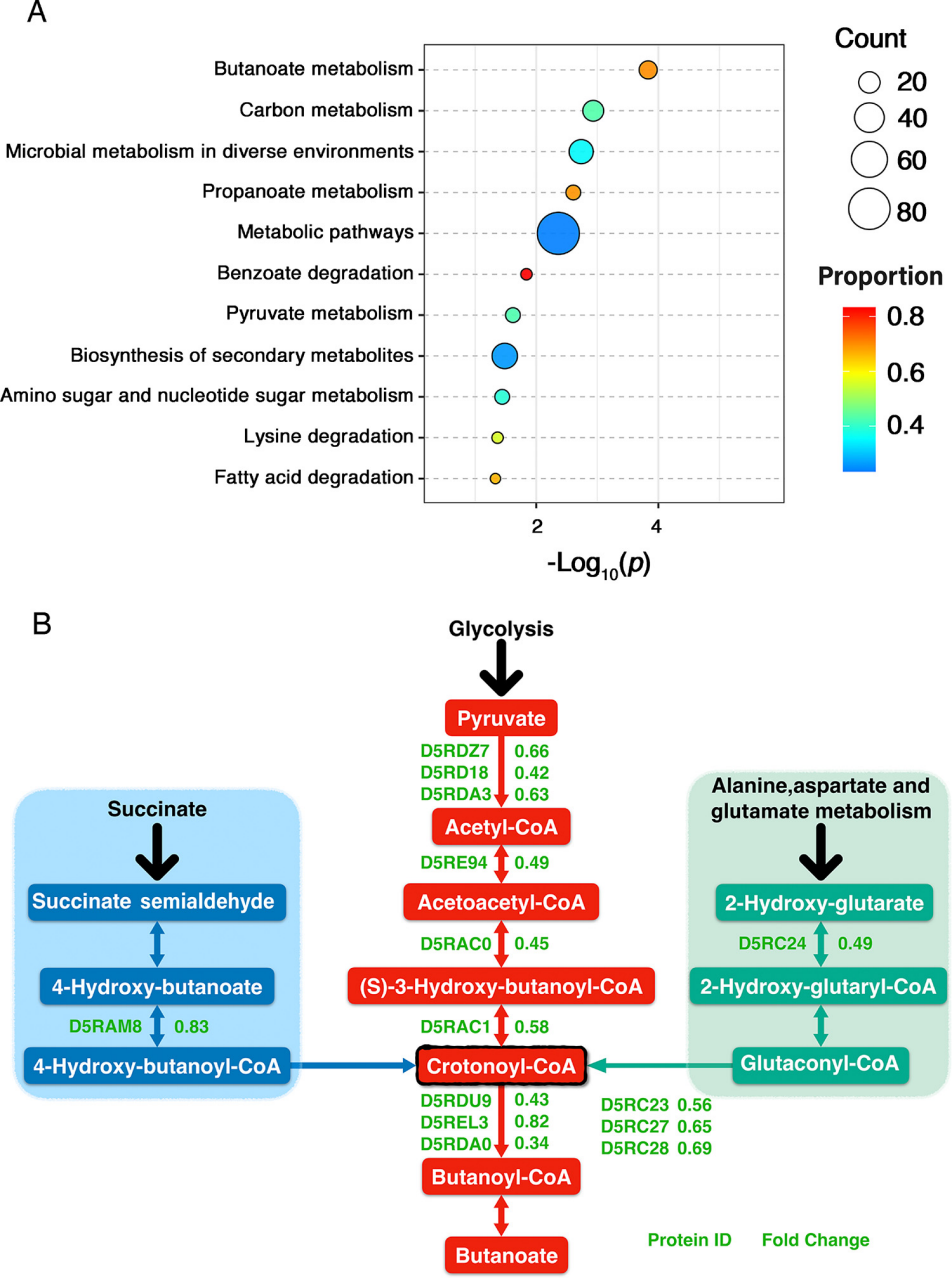
KEGG enrichment analysis of differentially enriched proteins in aOMVs/nOMVs. (A) KEGG enrichment map of downregulated proteins. (B) Butanoate metabolism KEGG pathways. The horizontal axis value is a negative logarithmic transformation of significant *P* values (*P* values of <0.05).

The downregulated differentially expressed proteins were enriched mainly in 12 KEGG metabolic pathways (Fig. 5A), including butyrate metabolism, carbon metabolism, microbial metabolism in different environments, propionate metabolism, benzoate metabolism, and pyruvate metabolism. Butyric acid and propionic acid are short-chain fatty acids (SCFAs), which are metabolites produced by the intestinal flora through the fermentation of dietary fiber (43). Butyrate has received extensive attention in recent years because it plays a key role in maintaining intestinal homeostasis and epithelial integrity and is a major energy source for colon cells (44). Butyrate directly affects host gene expression by inhibiting histone deacetylases and interfering with proinflammatory signals such as NF-κB. Previous studies have shown that a high concentration of butyrate can inhibit colorectal cancer development (45). F. nucleatum releases a large amount of short-chain fatty acids, including butyrate (46), during its growth, but little is known about the regulatory mechanism of butyrate metabolism in bacterial cells in the tumor environment. Four major butyrate synthesis pathways exist in gut microbes (47), namely, the pyruvate-acetyl-CoA, glutarate, 4-aminobutyric acid, and lysine pathways. Among them, the pyruvate-acetyl-CoA pathway is linked to glycolysis and is the most important butyrate synthesis pathway. All butyrate synthesis pathways converge at the central energy-generating step in which crotonyl-CoA can conserve energy during the conversion of crotonyl-CoA to butyl-CoA. Based on the aOMV/nOMV differentially expressed protein data, the differentially expressed proteins were enriched mainly in three butyrate synthesis pathways: the pyruvate-acetyl-CoA, glutarate, and 4-aminobutyrate pathways (Fig. 5B and Table S4). In the pyruvate-acetyl-CoA pathway, the expression levels of six proteins related to butyrate synthesis, pyruvate synthase (https://www.uniprot.org/, D5RDA3 and D5RD18), formate C-acetyltransferase (D5RDZ7), acetyl-CoA C-acetyltransferase (D5RE94), 3-hydroxyacyl-CoA dehydrogenase (D5RAC0), and enoyl-CoA hydratase (D5RAC1), were downregulated, among which pyruvate synthase (D5RD18) was significantly downregulated by 0.42-fold. The downregulation of pyruvate synthase can inhibit the oxidative decarboxylation of pyruvate to form acetyl-CoA, reducing the amount of acetyl-CoA entering the butyrate metabolic pathway. Four proteins in the glutarate pathway, glutaconate CoA transferase subunit B (D5RC24), oxaloacetate decarboxylase gamma chain (D5RC28), glutaconyl-CoA decarboxylase subunit alpha (D5RC23), and putative glutaconyl-CoA decarboxylase subunit gamma (D5RC27), were significantly downregulated. Only 4-hydroxybutyrate coenzyme A transferase (D5RAM8) was downregulated in the 4-aminobutyric acid pathway. Furthermore, the synthesis of crotonyl-CoA was inhibited in the above-mentioned three butyrate synthesis pathways. In conclusion, butyrate synthesis in F. nucleatum was significantly downregulated at pH 6.0.

### Changes in pH affect the expression of virulence factors.

Outer membrane vesicles are nanosized double-membrane vesicles secreted by bacterial cells. They are spherical and can transmit parent cytotoxic proteins into host cells, thereby inducing the host inflammatory response and causing host death. The type V-type secretion systems is an important pathway for the secretion of virulence factors in Gram-negative bacteria, and components of this system are called autotransporters. As virulence factors, autotransporters participate in various physiological processes such as adhesion, aggregation, infection, biofilm formation, serum resistance, and toxicity. To explore the effects of environmental pH changes on virulence factors, the expression levels of autotransporters and MORN2 domain-containing proteins in aOMVs and nOMVs were compared and analyzed. A total of 29 autotransporters (Table 1) were identified in two outer membrane vesicles, most of which belonged to T5aSS, a monomeric autotransporter. Through functional prediction analysis, these proteins were divided into serine proteases, adhesion proteins, and unknown proteins. Compared with nOMVs, seven T5aSSs in aOMVs were upregulated: two were autotransporters with serine-type endopeptidase activity (D5RA65 and D5RB84), four were autotransporters with adhesion function (D5RD74, D5REI9, D5RD69, and D5RBW2), and one was an autotransporter of unknown function associated with the formation of outer membranes. The autotransporters with adhesion function are all homologous to Fap2. Previous studies reported that Aim1 (D5RD74) can induce Jurkat cell apoptosis and inhibit the immune response. However, the functions of the other three adhesin homologs are still unclear (48). Seven T5bSS proteins were identified in both aOMVs and nOMVs. T5bSS, also known as the two-partner secretion system (TPS), is composed of two proteins: the secreted TpsA cargo protein and the TpsB chaperone transporter (49). Compared with nOMVs, five T5bSS proteins were upregulated in aOMVs, among which D5REQ7, D5RE85, and D5RF52 are filomagglutinin family proteins and act as effector proteins, while D5RCX9 and D5RE06 are polypeptide transport-associated (POTRA) domain proteins and belong to the TpsB chaperone transporter protein family. The function and mechanism of autotransporter type 5b in the virulence of F. nucleatum have not yet been reported (50). Meanwhile, a partial trimeric T5dSS protein and a monomeric T5dSS protein were also detected in aOMVs and nOMVs. Among the T5cSS proteins, only the D5RBQ0 protein level was increased. D5RBQ0 is a protein containing the YadA-like domain. Umaña et al. hypothesized previously that D5RBQ0 could be a nonfunctioning T5cSS adhesin (51). Only one T5dSS protein (D5RFI3) was detected in both aOMVs and nOMVs, and its expression levels were comparable in aOMVs and nOMVs. In conclusion, the protein levels of autotransporters associated mainly with adhesion and protease functions were upregulated in aOMVs.

**TABLE 1:** The type V secreted autotransporters identified in both aOMVs and nOMVs

Protein ID	Gene	Protein name or description	Threshold^*b*^	Type
D5REI9	HMPREF0397_1624	Uncharacterized protein^*a*^	Up	A
D5RD74	HMPREF0397_1159	Autotransporter beta-domain protein^*a*^	Up	A
D5RA65	HMPREF0397_0100	Peptidase_S8 domain-containing protein^*a*^	Up	A
D5REX7	HMPREF0397_1762	Autotransporter beta-domain protein	Up	A
D5RD69	HMPREF0397_1154	Uncharacterized protein^*a*^	Up	A
D5RBW2	HMPREF0397_0697	Autotransporter beta-domain protein	Up	A
D5RB84	HMPREF0397_0469	Peptidase_S8 domain-containing protein^*a*^	Up	A
D5RFW7	HMPREF0397_2102	Outer membrane autotransporter barrel domain protein^*a*^	Down	A
D5RD68	HMPREF0397_1153	Uncharacterized protein^*a*^	Down	A
D5RDJ8	HMPREF0397_1283	Autotransporter beta-domain protein	Down	A
D5RFX1	HMPREF0397_2106	Autotransporter beta-domain protein^*a*^	Down	A
D5REK4	HMPREF0397_1639	Outer membrane autotransporter barrel domain protein	NoSig	A
D5RDX9	HMPREF0397_1414	Uncharacterized protein^*a*^	NoSig	A
D5RE96	HMPREF0397_1531	Autotransporter beta-domain protein	NoSig	A
D5RBA3	HMPREF0397_0488	Autotransporter beta-domain protein	NoSig	A
D5RBA4	HMPREF0397_0489	Outer membrane autotransporter barrel domain protein	NoSig	A
D5REQ7	HMPREF0397_1692	Filamentous hemagglutinin family domain protein	Up	B
D5RCX9	*tpsB2*	POTRA domain protein, ShlB type	Up	B
D5RE85	HMPREF0397_1520	Filamentous hemagglutinin family domain protein^*a*^	Up	B
D5RE06	HMPREF0397_1441	POTRA domain protein, ShlB type^*a*^	Up	B
D5RF52	*tpsA2*	Filamentous hemagglutinin family domain protein	Up	B
D5RA64	HMPREF0397_0099	Hemolysin secretion/activation protein, ShlB/FhaC/HecB family^*a*^	Down	B
D5RCB6	HMPREF0397_0851	POTRA domain protein, ShlB type^*a*^	NoSig	B
D5RBQ0	HMPREF0397_0635	YadA-like domain protein	Up	C
D5RAW6	HMPREF0397_0351	Hep/Hag repeat protein	NoSig	C
D5RFW5	HMPREF0397_2100	Hep/Hag repeat protein^*a*^	NoSig	C
D5RET6	HMPREF0397_1721	Hep/Hag repeat protein^*a*^	NoSig	C
D5RBK6	HMPREF0397_0591	Hep/Hag repeat protein	NoSig	C
D5RFI3	HMPREF0397_1968	Phospholipase, patatin family	NoSig	D

a Fragment.

b Up indicates up-regulated differential protein. Down indicates down-regulated differential protein. NoSig indicates no change in protein expression.

**FIG 6:**
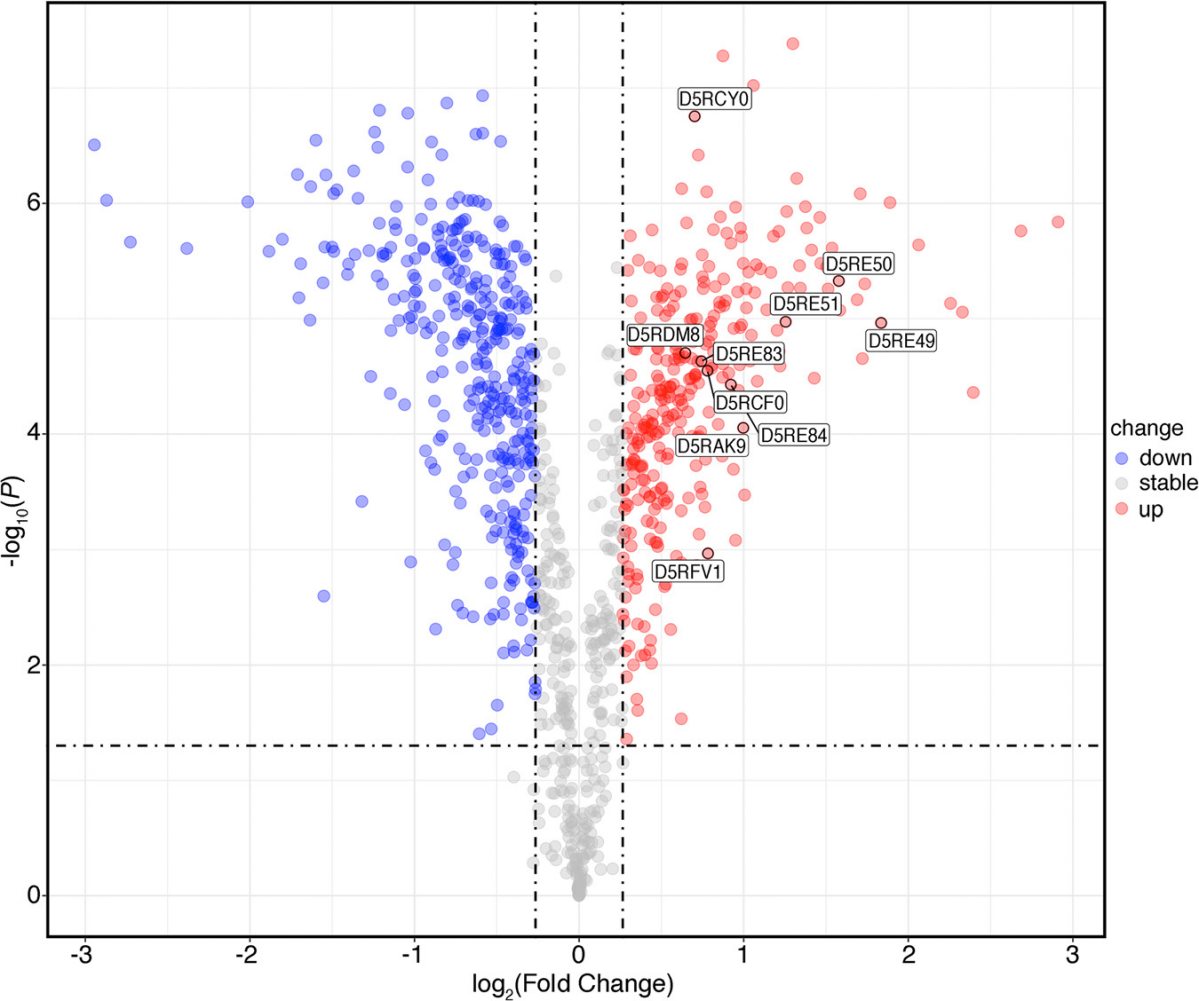
Volcano plot where the 10 most significantly upregulated MORN domain-containing proteins are labeled. The horizontal axis represents the logarithmic transformation based on 2 of the fold change, and the vertical axis represents the logarithmic transformation based on 10 of the *P* value. The red dots and blue dots represent proteins with a fold change of >1.2 (upregulation of >1.2-fold or downregulation of <0.83-fold, respectively) and a *P* value of <0.05, and the gray dots represent proteins with no significant difference.

A total of 23 MORN2 domain-containing proteins were identified in aOMVs and nOMVs, and 16 MORN2 domain-containing proteins were upregulated in aOMVs (Table 2). A volcano plot was used to visualize the distribution of the 10 most significantly upregulated (FC of >1.5) MORN2 domain-containing proteins (Fig. 6). By comparing whole-genome sequences, Manson McGuire et al. found that while F. nucleatum bacteria are actively invading, they possess a large number of proteins containing MORN2 domains, and those researchers proposed that proteins containing MORN2 domains may have functions in virulence, invasion, and adhesion (52). The MORN2 domain-containing protein D5RCF0 was recently identified as a key element in actively invading F. nucleatum species (53). Umaña et al. suggested that the genomes of MORN2 domain-containing proteins are clustered near the genes of the T5aSS autotransporter adhesin; thus, MORN2 domain-containing proteins can enhance bacterial adhesion and active invasion capabilities (51).

**TABLE 2:** The MORN2 domain-containing proteins identified in both aOMVs and nOMVs

Protein ID	Gene	Protein description	Threshold
D5RE49	HMPREF0397_1484	MORN repeat protein	Up
D5RE50	HMPREF0397_1485	MORN repeat protein	Up
D5RE51	HMPREF0397_1486	MORN repeat protein	Up
D5RAK9	HMPREF0397_0244	MORN repeat protein	Up
D5RE84	HMPREF0397_1519	MORN repeat protein	Up
D5RFV1	HMPREF0397_2086	MORN repeat protein	Up
D5RCF0	HMPREF0397_0885	MORN repeat protein	Up
D5RE83	HMPREF0397_1518	MORN repeat protein	Up
D5RCY0	HMPREF0397_1065	MORN repeat protein	Up
D5RDM8	HMPREF0397_1313	MORN repeat protein	Up
D5RBF7	HMPREF0397_0542	MORN repeat protein	Up
D5RAX9	HMPREF0397_0364	MORN repeat protein	Up
D5RDV8	HMPREF0397_1393	MORN repeat protein	Up
D5RD88	HMPREF0397_1173	MORN repeat protein	Up
D5RB13	HMPREF0397_0398	MORN repeat protein	Up
D5RFV0	HMPREF0397_2085	MORN repeat protein	Up
D5RE34	HMPREF0397_1469	MORN repeat protein	NoSig
D5RBK3	HMPREF0397_0588	MORN repeat protein	NoSig
D5RAX6	HMPREF0397_0361	MORN repeat protein	NoSig
D5RAX8	HMPREF0397_0363	MORN repeat protein	NoSig
D5RAI0	HMPREF0397_0215	MORN repeat protein	NoSig
D5RB12	HMPREF0397_0397	MORN repeat protein	NoSig
D5RCM2	HMPREF0397_0957	MORN repeat protein	Down

## DISCUSSION

This study demonstrated that F. nucleatum can grow and secrete outer membrane vesicles under neutral or acidic conditions. Compared with neutral conditions, F. nucleatum released more OMVs under acidic conditions, and the protein composition of OMVs was changed greatly. The possible reason for the above-mentioned results is that bacterial cells relieve the stress caused by low pH by releasing more outer membrane vesicles to improve their survival probability.

There are a few reports on the protein composition of F. nucleatum OMVs and no reports on the protein composition changes of F. nucleatum OMVs under different pH conditions. Therefore, we used acidic cysteine to adjust the initial pH of TSPC medium (3% tryptic soy broth [TSB] supplemented with 1% Bacto peptone [TSP]) to 6.0 to mimic the tumor microenvironment pH and performed comparative proteomic and metabolomics analyses. To the best of our knowledge, this is the first comparative analysis of the protein composition and function of F. nucleatum OMVs based on TMT quantitative proteomics technology. To improve the purity of the outer membrane vesicle samples, we referred to a method described previously by Wagner et al. and used an iodixanol density gradient centrifugation technique to purify the outer membrane vesicles to reduce the interference of non-outer membrane vesicle proteins in the analysis (54). We found that the proteins in the F. nucleatum OMVs were derived mainly from the cell membrane of the parent cells, followed by the cytoplasm. However, Liu et al. reported that half of the proteins in F. nucleatum OMVs belong mainly to cytoplasmic matrix proteins, followed by outer membrane proteins (37). We believe that this difference is unlikely to be due to protein contamination since the outer membrane vesicles were purified by the iodixanol density gradient centrifugation method in both studies, so the difference may be due to differences in the subspecies and culture conditions. Based on TMT quantitative proteomics technology, we detected a total of 991 proteins in aOMVs and nOMVs, including the known virulence proteins FadA, Fap2, and FomA and putative MORN2 domain-containing and Aim1 virulence proteins. These proteins are classified as outer membrane proteins, enzymes, and proteins involved in transport, indicating that F. nucleatum OMVs have multiple potential functions. We found that there were 666 differentially expressed proteins in aOMVs and nOMVs using TMT-based quantitative MS proteomics analysis. Compared to nOMVs, 306 proteins were upregulated and 360 proteins were downregulated. This result shows that approximately 70% of the expression of OMV proteins is altered under acidic conditions.

Pathogenic bacteria use a variety of virulence proteins to infect host cells to achieve long-term survival and reproduction in the host. Virulence proteins are either exposed to the surface of the bacterial cells or secreted to the outside of the cells in the form of secreted proteins. Virulence proteins induce tumorigenesis by interacting with specific macromolecular receptors of host cells, causing pathological damage or inducing host immune responses. F. nucleatum can transmit virulence proteins by secreting outer membrane vesicles, and a variety of virulence proteins contained in outer membrane vesicles are involved in the communication between bacteria and the host. Adhesion is crucial in bacterial pathogenesis, especially during bacterial colonization and dissemination. Manson McGuire et al. found by analyzing the whole-genome sequence of F. nucleatum that genes encoding MORN2 domain-containing proteins are abundant in actively invasive F. nucleatum bacteria (52). They also found that genes encoding MORN2 domain-containing proteins are located near genes encoding T5aSS proteins and adhesins such as FadA and RadD in the genome. It has been speculated that proteins containing MORN2 domains can enhance adhesion and invasiveness (51). In addition, genes encoding MORN2 domains are clustered with genes encoding membrane-associated pathogenic factors (such as OmpA proteins) and virulence factors (proteins containing chorismate mutase domains), so MORN2 domain-containing proteins may have toxic effects (55, 56). We found multiple MORN2 domain-containing proteins in both nOMVs and aOMVs, and compared to nOMVs, approximately 70% of the MORN2 domain-containing proteins were upregulated in aOMVs. Thus, we speculate that the adhesion and invasion abilities of F. nucleatum are enhanced under acidic conditions. An adhesion protein containing the YadA-like domain has also been found in nOMVs and aOMVs. YadA-like domain protein levels were upregulated 2-fold in aOMVs compared to nOMVs. The YadA-like domain protein is a key virulence protein in Yersinia pseudotuberculosis, mediating adhesion to host cells by binding to extracellular matrix components (57). At present, there are no specific experimental results to prove that MORN2 domain-containing proteins enhance the adhesion and invasion of bacteria, and an in-depth study of the function and mechanism of MORN2 domain-containing proteins would be of great significance for revealing the mechanism of the interaction between F. nucleatum and the host and for providing new clues for preventing and treating colorectal cancer (15).

F. nucleatum is unique in that it achieves infection of host cells through a type V secretion system, thereby threatening the survival of host cells. F. nucleatum secretes autotransporters through the type V secretion system, which are outer membrane proteins or secreted proteins that can be divided into five classes (Va to Ve) according to their domains. Most autotransporters are known or predicted virulence factors. Autotransporters display multiple virulence-related functions, including adhesion, invasion, and cytotoxicity. A total of 29 autotransporters were identified in nOMVs and aOMVs, and 13 autotransporters were upregulated in aOMVs compared to nOMVs. Interestingly, multiple upregulated autotransporters (D5REI9, D5RD69, and D5RBW2) show homology to the identified virulence factor Fap2 (51), and it has been speculated that they may have functions similar to those of the Fap2 protein and may be involved in various pathogenic pathways such as the pathway for binding with colorectal cancer cells (58), the induction of lymphocyte apoptosis pathway, the natural killer cell inhibitory receptor T cell immunoglobulin and ITIM domain interaction pathway (59), and the immune escape pathway, etc. Whether F. nucleatum outer membrane vesicles function by altering the tumor immune microenvironment or by direct contact with tumor cells will be investigated in future work.

Using TMT quantitative proteomics technology and bioinformatics analysis, we found that more than 70% of MORN2 domain-containing proteins and approximately 50% of type V autotransporter proteins were upregulated in F. nucleatum OMVs under acidic conditions. Currently, the functions and mechanisms of action of MORN2 domain-containing proteins and most type V autotransporters in adhesion, invasion, and toxicity have not been validated. In the future, we will focus on the above-mentioned MORN2 domain-containing proteins and type V autotransporter proteins and clarify their relationship with adhesion, invasion, and toxicity by constructing gene mutants and combining *in vivo* and *in vitro* biological experiments to develop a new method to prevent and treat colorectal cancer and other diseases.

## MATERIALS AND METHODS

### Bacterial strain and growth conditions.

F. nucleatum ATCC 23726 was purchased from the American Type Culture Collection. F. nucleatum was cultured anaerobically (93% N_2_, 5% CO_2_, 2% H_2_) at 37°C in TSPC medium, which consisted of 3% tryptic soy broth (Qingdao Hope Bio-Technology) and 1% Bacto peptone (Life Technologies Corporation) supplemented with 2% (vol/vol) freshly made l-cysteine (0.13 g/mL) for creating normal intestinal pH (pH 7.0). To create a tumor microenvironment at pH 6.0, TSPC medium supplemented with 2% (vol/vol) freshly made l-cysteine hydrochloride monohydrate (0.18 g/mL) was used. The bacteria were harvested when the optical density at 600 nm reached 1.0.

### Isolation and purification of outer membrane vesicles.

F. nucleatum OMVs were isolated as follows. Briefly, bacteria were cultured as described above. Following the pelleting of bacterial cells by centrifugation at 8,500 × *g* for 20 min at 4°C (type J2-MC rotor; Beckman Coulter) to remove all bacterial cells in the cultures, the supernatants were filtered using a 0.22-μm sterile filter (Sigma) to remove parental bacterial debris and other contaminants. The supernatant was further concentrated 50-fold by using a 0.2-μm hollow-fiber membrane (catalog no. xh-110-pp; XinHui Membrane Technology, China). Next, the OMVs were pelleted from the supernatant by ultracentrifugation at 200,000 × *g* for 4 h at 4°C (type S110AT-2084 rotor; Hitachi). The pellets containing OMVs were resuspended in sterile phosphate-buffered saline (PBS). The resuspended OMVs were purified by density gradient centrifugation using OptiPrep (60% [wt/vol] iodixanol; Serumwerk Bernburg AG). The OptiPrep solution was diluted with HEPES (Sangon Biotech)-buffered saline (0.85% [wt/vol] NaCl, 10 mM HEPES-NaOH [pH 7.4]) to 40%, 35%, 30%, 25%, 20%, and 15% (vol/vol) OptiPrep densities. The discontinuous gradients were loaded in 500-μL increments in decreasing concentrations on top of the prior layer of a 5-mL ultracentrifuge tube. Next, 300 μL of concentrated OMVs was added to the top layer. The prepared tubes were centrifuged at 135,000 × *g* for 16 h at 4°C (type S110AT-2084 rotor; Hitachi). Each 300-μL fraction was carefully collected by pipetting from top to bottom. The OMV content in each fraction was analyzed by 12% SDS-PAGE. OMV-enriched fractions were collected, mixed well with PBS with at least 20-fold the sample volume, pooled in ultracentrifuge tubes, and ultracentrifuged at 200,000 × *g* for 4 h at 4°C (type S110AT-2084 rotor; Hitachi) to remove the OptiPrep solution. The purified OMVs were resuspended in 100 μL of sterile PBS buffer and stored in aliquots at −80°C for future use.

### Transmission electron microscopy.

Transmission electron microscopy (TEM) was used to visualize the outer membrane vesicle samples. The resuspended samples were added dropwise to 200-mesh grids (Beijing Zhongjingkeji Technology) and incubated at room temperature for 10 min, the grids were then negatively stained with 2% phosphotungstic acid (SPI-chem) for 3 min, and the remaining liquid was removed by using filter paper. Next, the cells were observed with a transmission electron microscope (HT7700; Hitachi, Japan).

### Scanning electron microscopy.

The OMVs were fixed with 2.5% glutaraldehyde in PBS (Sangon Biotech, China). After washing with PBS, the dried samples were coated with gold by a sputter coater (SBC-12; KYKY, China) using the physical vapor deposition method. The prepared samples were examined by scanning electron microscopy (SEM) (S-4800; Hitachi, Japan).

### Nanoparticle tracking analysis.

The size distribution and concentration of outer membrane vesicles were measured by nanoparticle tracking analysis (NTA) (Particle Metrix, Meerbusch, Germany). The vesicle samples were appropriately diluted using 1× PBS buffer (Sangon Biotech) to measure the particle size and concentration. NTA measurements were recorded and analyzed at 11 positions. A polystyrene particle of 100 nm was used to calibrate the ZetaView system.

### Tryptic digestion.

The OMV samples were rethawed at 4°C while grinding with liquid nitrogen, homogenized in 100 μL SDT lysis buffer (4% SDS, 100 mM Tris, 10 mM dithiothreitol [DTT]), and incubated for 3 min at 95°C. Next, the samples were sonicated for 2 min at 4°C. The total protein was collected by centrifugation at 16,000 × *g* (5424R centrifuge; Eppendorf) for 20 min at 4°C, and the protein concentration was determined using a bicinchoninic acid (BCA) protein assay kit (Bio-Rad, USA). An OMV sample corresponding to 100 μg of protein was dropped into an ultrafiltration tube (trapped relative molecular mass of 10 kDa) and centrifuged at 13,000 × *g* for 30 min after the addition of 200 μL of UA buffer (8 M urea, 150 mM Tris-HCl [pH 8.0]). After the supernatant was discarded, 100 μL indole acetic acid (IAA) (50 mM IAA in UA buffer) was added, and the samples were mixed lightly, incubated at room temperature for 30 min in the dark, and then centrifuged at 12,000 × *g* for 10 min. The samples were digested with trypsin (6 μg trypsin in 40 μL NH_4_HCO_3_ buffer) overnight at 37°C. The samples were acidified with a final concentration of 0.5% trifluoroacetic acid to quench the enzyme activity and precipitate sodium deoxycholate, which was subsequently removed by centrifugation. The supernatants were dried in a vacuum centrifuge and redissolved in 0.1% trifluoroacetic acid.

### TMT labeling of peptides.

Peptides were labeled with TMT reagents according to the manufacturer’s instructions (Thermo Fisher Scientific). Each aliquot (100 μg of peptide equivalent) was reacted with one tube of TMT reagent. After the sample was dissolved in 100 μL of a 0.05 M triethylammonium bicarbonate (TEAB) solution (pH 8.5), the TMT reagent was dissolved in 41 μL of anhydrous acetonitrile. The mixture was incubated at room temperature for 1 h. Next, 8 μL of 5% hydroxylamine was added to the sample, and the sample was incubated for 15 min to quench the reaction. The multiplex-labeled samples were pooled and lyophilized. To increase the depth of protein identification, peptide fractionation was performed using a Pierce high-pH reversed-phased peptide fractionation kit (Thermo Fisher Scientific) according to the manufacturer’s instructions (60). Peptides from each fraction were dried and redissolved with 0.1% formic acid for LC-MS analysis.

### LC-MS/MS analysis and database search.

The appropriate amounts of peptides for each sample were separated by chromatography using the Easy nLC 1200 chromatography system (Thermo Scientific). The peptides were separated by DDA (data-dependent acquisition) mass spectrometry with a Q-Exactive HF-X mass spectrometer (Thermo Scientific). The resulting LC-MS/MS raw file was imported into the Sequest HT search engine in Proteome Discoverer software (version 2.4; Thermo Scientific) for database retrieval.

### Bioinformatics analysis.

Analysis of bioinformatics data was carried out with Perseus software, Microsoft Excel, and R statistical computing software. Significantly differentially expressed proteins were screened with cutoffs of a fold change ratio of >1.20 or <0.83 and a *P* value of <0.05. Expression data were grouped by hierarchical clustering according to the protein level. To annotate the sequences, information was extracted from the UniProtKB/Swiss-Prot, KEGG, and GO databases. GO and KEGG enrichment analyses were carried out using Fisher’s exact test, and false discovery rate (FDR) correction for multiple testing was also performed. GO terms were grouped into three categories: biological processes (BPs), molecular functions (MFs), and cellular components (CCs). Enriched GO and KEGG pathways were nominally statistically significant at a *P* value of <0.05. The construction of protein-protein interaction (PPI) networks was also conducted using the STRING database with Cytoscape software.

### Data availability.

The mass spectrometry proteomics data have been deposited to the ProteomeXchange Consortium (http://proteomecentral.proteomexchange.org) via the iProX partner repository (61) with the data set identifier PXD037520.
