# Supplementary figures and images for: Quantitative Proteomic Analysis of Outer Membrane Vesicles from Fusobacterium nucleatum Cultivated in the Mimic Cancer Environment
Source: Microbiol Spectr. 2023 Jun 21;11(4):e00394-23. doi: 10.1128/spectrum.00394-23 (PMC10434195; doi:10.1128/spectrum.00394-23)

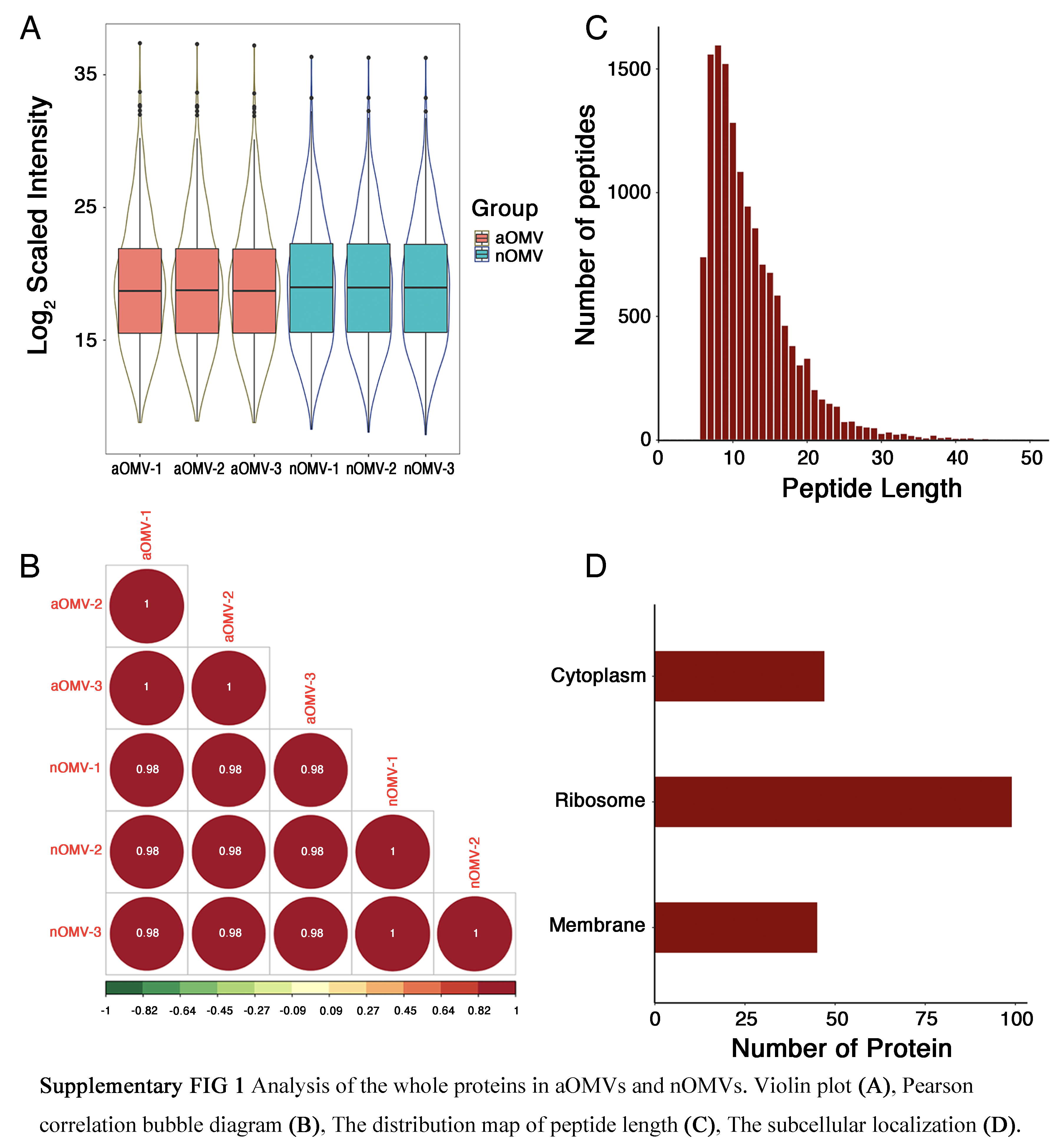

Supplement: Supplemental file 1 — Fig. S1. Download spectrum.00394-23-s0003.tif, TIF file, 7.2 MB [file spectrum.00394-23-s0003.tif]

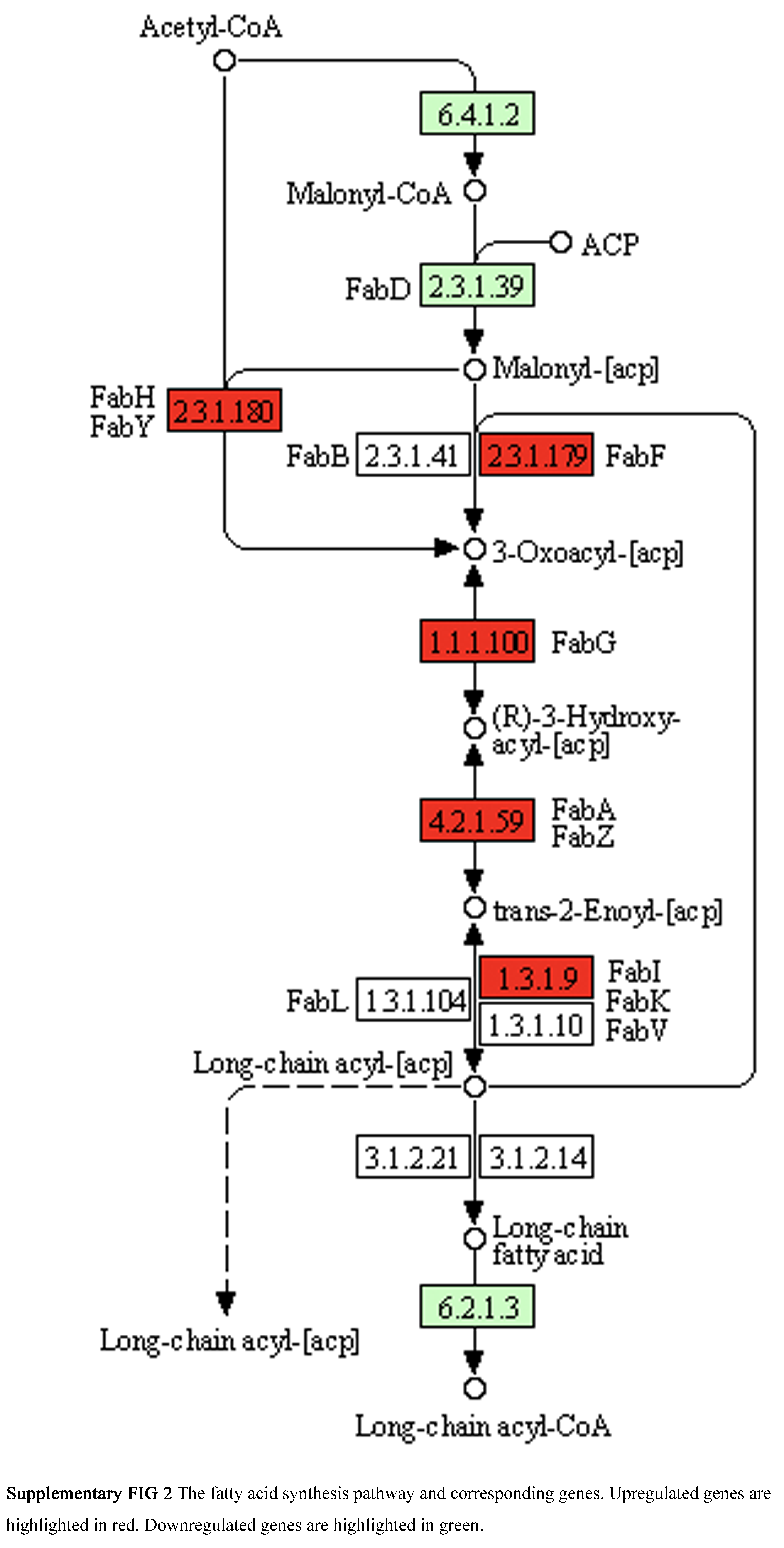

Supplement: Supplemental file 2 — Fig. S2. Download spectrum.00394-23-s0004.tif, TIF file, 9.6 MB [file spectrum.00394-23-s0004.tif]
