# Supplementary material for: Quantitative Proteomic Analysis of Outer Membrane Vesicles from Fusobacterium nucleatum Cultivated in the Mimic Cancer Environment
Source: Microbiol Spectr. 2023 Jun 21;11(4):e00394-23. doi: 10.1128/spectrum.00394-23 (PMC10434195; doi:10.1128/spectrum.00394-23)
Supplement: Supplemental file 3 — Tables S1 and S4. Download spectrum.00394-23-s0005.docx, DOCX file, 0.02 MB [file spectrum.00394-23-s0005.docx]

| Supplementary Table 1 | | | | | |
| --- | --- | --- | --- | --- | --- |
| Statistics on the protein identification results | | | | | |
| Identification Results | Number of MS/MS | Number of PSM | Unique Petide | Protein Groups | Quantified Protein |
| Total | 549010 | 78986 | 14039 | 991 | 991 |

| Supplementary Table 4 | | | |
| --- | --- | --- | --- |
| Differential proteins in three butyric acid synthesis pathways in aOMV/nOMV | | | |
| Accession | Gene Symbol | Protein Name | Threshold |
| D5RAM8 | cat | 4-hydroxybutyrate coenzyme A transferase | Down |
| D5RDU9 | bcd2 | Acyl-CoA dehydrogenase, C-terminal domain protein | Down |
| D5REL3 | acdA | Acyl-CoA dehydrogenase, C-terminal domain protein | Down |
| D5RDA0 | bcd | Rubredoxin | Down |
| D5RC28 | HMPREF0397_0763 | Oxaloacetate decarboxylase gamma chain | Down |
| D5RC23 | gcdA | Glutaconyl-CoA decarboxylase subunit alpha | Down |
| D5RC27 | HMPREF0397_0762 | Putative glutaconyl-CoA decarboxylase subunit gamma | Down |
| D5RAC1 | crt | Enoyl-CoA hydratase/isomerase family protein | Down |
| D5RAC0 | hbd | 3-hydroxyacyl-CoA dehydrogenase, NAD binding domain protein | Down |
| D5RC24 | gctB | Glutaconate CoA-transferase subunit B | Down |
| D5RE94 | atoB | Acetyl-CoA C-acetyltransferase | Down |
| D5RDZ7 | pflB | Formate C-acetyltransferase | Down |
| D5RD18 | nifJ | Pyruvate synthase | Down |
| D5RDA3 | nifJ | Pyruvate synthase | Down |
